# Revealing ultralarge and localized elastic lattice strains in Nb nanowires embedded in NiTi matrix

**DOI:** 10.1038/srep17530

**Published:** 2015-12-02

**Authors:** Ketao Zang, Shengcheng Mao, Jixiang Cai, Yinong Liu, Haixin Li, Shijie Hao, Daqiang Jiang, Lishan Cui

**Affiliations:** 1 Beijing Key Lab of Microstructure and Property of Advanced Materials, Beijing University of Technology, Beijing, 100124, China; 2 School of Mechanical and Chemical Engineering, The University of Western Australia, Crawley, WA 6009, Australia; 3 GRIKIN Advanced Materials Co. Ltd., Beijing, 102200, China; 4 Department of Materials Science and Engineering, China University of Petroleum-Beijing, Beijing, 102249, China

## Abstract

Freestanding nanowires have been found to exhibit ultra-large elastic strains (4 to 7%) and ultra-high strengths, but exploiting their intrinsic superior mechanical properties in bulk forms has proven to be difficult. A recent study has demonstrated that ultra-large elastic strains of ~6% can be achieved in Nb nanowires embedded in a NiTi matrix, on the principle of lattice strain matching. To verify this hypothesis, this study investigated the elastic deformation behavior of a Nb nanowire embedded in NiTi matrix by means of *in situ* transmission electron microscopic measurement during tensile deformation. The experimental work revealed that ultra-large local elastic lattice strains of up to 8% are induced in the Nb nanowire in regions adjacent to stress-induced martensite domains in the NiTi matrix, whilst other parts of the nanowires exhibit much reduced lattice strains when adjacent to the untransformed austenite in the NiTi matrix. These observations provide a direct evidence of the proposed mechanism of lattice strain matching, thus a novel approach to designing nanocomposites of superior mechanical properties.

Elastic lattice deformation of crystalline solids is of interest because it is the main mechanism of load carrying (i.e., strength) of solids. In addition, many of the physical and chemical properties of crystalline solids depend on the valence electron states, which may be influenced by large lattice strains. Therefore, it is of our keen interest to achieve high elastic strains in solids. The theoretical limits of elastic strains of nanomaterials have been predicted to be as high as 8%^1^,^2^,^3^,^4^, as verified in single crystal Cu nanowires by means of *in situ* TEM measurement during tension^2^ and in single alumina nanowhiskers by means of nanomechanics testing in bending^4^. However, bulk metals generally exhibit elastic strains much less than the theoretical limits, typically below 1%, largely due to structural defects which trigger premature failure of the materials. Considering the above, it has become a highly contested concept to compose nanomaterials into metallic alloy matrices to create bulk materials of superior mechanical properties^5^. However, despite the numerous nanowire-metal matrix composite systems attempted, few have been able to harness the exceptional intrinsic mechanical properties of nanomaterials, as manifested by the generally low elastic strains achieved in the embedded nanomaterials, typically below 1.5%^6^,^7^,^8^,^9^.

Scientists have attributed this failure to the less-than-ideal microstructures of the composites, such as non-uniform distribution of the nanowires in the matrix, lack of alignment in one (axial) direction, or poor bonding with the matrix^5^,^10^. Motivated by this understanding, more efforts have been made to overcome these obstacles, and some improvements have been achieved^8^,^11^,^12^. A typical example is the Nb nanowire-Cu matrix composite, in which the nanowires are well dispersed and well aligned, and are with strong interfacial bonding. The maximum elastic strain achieved in the Nb nanowires in this composite is 1.75%^8^,^11^, still well below the expected intrinsic capabilities of the nanowires^5^,^10^.

To tackle this problem, Cui *et al.* recently designed a new class of metallic nanocomposites in which Nb nanowires are embedded in a NiTi shape memory alloy matrix^13^. The NiTi matrix deforms via a martensitic transformation between a B2 phase and a B19′ monoclinic martensite^14^. Differing from all other conventional metallic matrices attempted before, which deform by the mechanism of plasticity via dislocation activity^6^,^7^,^8^,^11^, this composite system provided a novel nanomechanics system at the atomic level of lattice strain matching between the phase transformation lattice distortion of the martensite and uniform elastic strains of the nanowires. Using *in situ* synchrotron X-ray diffraction analysis during tensile deformation, ultra-large elastic lattice strains of ~6% were measured in the Nb nanowires embedded in the NiTi matrix^15^,^16^. It needs to be pointed out that the elastic lattice strains measured by X-ray diffraction method are average values from the many Nb nanowires within the detection volume of diffraction (millimeters), whereas the strain matching hypothesis predicts that the elastic strains of a nanowire may vary spatially along its length in response to variations in the phase structure (martensite or austente) of the NiTi matrix, typically at a sub-micron scale. In addition, it is known that the transformation strains of the B19′ martensite in NiTi range from 3.7% to 10.6% depending on the crystal orientation^14^,^17^. In this regard, whereas providing a strong factual support, the X-ray diffraction analysis was unable to provide direct evidence to verify the lattice strain matching mechanism. To address this uncertainty, this study was conducted to investigate the elastic deformation behaviors of Nb nanowire embedded in a NiTi matrix at the atomic scale by using *in situ* TEM measurement during tensile deformation, with a particular focus on the interactions between the Nb nanowire and the NiTi matrix during stress-induced B2→B19′ martensitic transformation to elucidate the direct evidence of lattice strain matching between the nanowire and the matrix^18^,^19^,^20^,^21^,^22^,^23^. Deformation of nanomaterials and simultaneously observe the evolution of microstructure at the atomic scale has been a long standing challenge. These challenge was successfully resolved by designing an apparatus as schematically shown in Fig. 1 for tensile deformation of nanomaterials in TEM. The tensile apparatus consists of two bimetallic arms, which bend away from each other to create a tension upon heating, as illustrated in Fig. 1c. Four microbeams of ~0.27 × 1.0 μm^2^ (Fig. 1b) were cut from the plate using a Helios Nanolab 600i focused ion beam milling equipment integrated in a high resolution scanning electron microscope. For *in situ* observation under TEM, the heating rate was controlled to generate a strain rate of ~4 × 10^−4^/s to the sample. The tensile deformation can be approximately treated as uniaxial tension since the elongations of the microbeams (<200 nm) are very small compared to the length of the bimetallic arms (~1.5 mm).

Figure 2 shows the microstructure of the Nb/NiTi composite fabricated. Figure 2a is a bright field TEM micrograph of the longitudinal section of the composite wire. The Nb nanowires are well aligned along the composite wire direction and uniformly distributed in the NiTi matrix, with diameters of 30–60 nm and an average inter-wire distance of ~50 nm. Figure 2b shows a selected area electron diffraction (SAED) pattern taken from the area shown in Fig. 2a. It is evident that both the Nb nanowires and the NiTi matrix had a strong [110] texture along the wire direction. Figure 2c shows a high resolution TEM (HRTEM) image of an interface between a Nb nanowire and the NiTi matrix. It is seen that the interface is clean and forms a perfect bonding between the two components.

The orientation variation of an individual Nb nanowire was further surveyed by taking SAED patterns at different regions along its length, as shown in Fig. 2d. Figures 2e–i show the SAED patterns corresponding to the regions indicated in Fig. 2d. The yellow lines indicate the 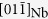 direction in the reciprocal space as determined at region (e), and the red lines represent the 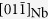 directions in each of the SAED patterns. It is seen that the Nb nanowire is a single crystal (within the observation range of ~1 μm) with an orientation misalignment of no more than 5° in different sections. SAED analysis was also conducted on several other Nb nanowires and similar observations were made.

The Nb/NiTi composite microplate was subjected to tensile deformation inside the TEM. Figure 3 shows TEM study of *in-situ* tensile deformation of a Nb/NiTi composite sample. The sample was fabricated by means of FIB and contained three parallel microbeams, and the deformation behavior of one of the microbeams was studied, as shown in Fig. 3a. The microbeam contained two Nb nanowires. Figure 3a shows the microbeam (the sample) under un-stressed state, from which the original d-spacing value of the atomic planes (the (110) planes) was determined, which is used as reference to calculate lattice strains of the microbeam under stress. The loading axis was parallel to the axial direction of the nanowires. Figure 3b shows an enlarged TEM image of a Nb nanowire as identified in Fig. 3a, Fig. 3c shows an area identified in Fig. 3b at a higher magnification, and Fig. 3i shows a filtered HRTEM image of the area identified in Fig. 3c. A few dislocations are seen in the Nb nanowire prior to deformation (Fig. 3i). From this HRTEM image the (110) plane d-spacing value under no stress was measured, to be d_(110)_ = 0.238 nm, as indicated in the micrograph. Upon heating from the room temperature (~20 °C) to 47 °C, the microbeam was gradually elongated to a strain of ~3.23%, as determined by measuring the length variation between the two reference points indicated in Fig. 3a,d. At this strain level, multiple martensite plates have nucleated in the NiTi matrix, as seen in Fig. 3d.

Figure 3e shows an enlarged view of region (e) identified in Fig. 3d. This region contains three martensite plates, marked as M_1_, M_2_ and M_3_. Figures 3f,g show high magnification images of the three corresponding regions inside the Nb nanowire as identified in Fig. 3e. Region **f** is near the tip of martensite plate M_1_, region **h** is in between martensite plates M_1_ and M_2_ (i.e., underneath the austenite), and region **g** is underneath the boundary between M_1_ and the austenite. Figure 3i–l are the filtered HRTEM images corresponding to the boxed regions of **i**-**l** indicated in Fig. 3c,f–h. The normal strains along the loading axis in the different regions were determined by measuring interplanar spacing changes, relative to Fig. 3i, of the (110)_Nb_ planes, which are perpendicular to the loading axis. The lattice strains are measured by measuring the relative dilation of the lattice plane spacings from these HRTEM images. The accuracy of lattice spacing measurement based on digital intensity spectrum of the TEM images is 0.0045 nm (pixel size), which gives to a relative accuracy of 0.27% for the strain determination when measuring over 7 ~ 10 lattice spacings. Also, to assure maximum accuracy and reliability of the measurements, the measurements were conducted away from dislocations and other structural defects. The deformation strains of the three regions are calculated to be 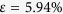 (region **f**), 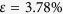 (region **g**) and 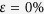 (region **h**). This observation clearly demonstrates that the elastic deformation of the Nb nanowire is highly inhomogeneous, apparently influenced by the discrete strain field of the matrix, which consists of the austenite and the martensite.

To further characterize the strain distribution in the Nb nanowire, (110)Nb lattice strains along the length of the Nb nanowire under martensite plates M1 and M2 were measured at small intervals (~1.6 nm). This was conducted by applying FFT to the area underneath the NiTi as identified by the dashed long box in Fig. 4a. The FFT-filtered image is shown at the bottom of Fig. 4a, with the lattice strain values determined also indicated. Figure 4b shows the lattice strain distribution as a function of distance from the start plane. The positions, at which the strains were measured, are consistent in (a) and (b). It is evident that the (110)_Nb_ lattice strain reached maximum (~8%) at under the tip of the martensite plate and minimum at the middle between two martensite plates. The maximum lattice strain measured in a localized region is higher than the average elastic strain (4.2–6.5%) macroscopically measured by using X-ray diffraction analysis^13^. Such ultra-large elastic strains, close to the theoretical limit of elasticity, have not been reported for nanowires embedded in a metallic matrix in the literature to date.

It is also evident in Fig. 4 that the elastic strain distribution along the length of the Nb nanowire was inhomogeneous^13^,^16^ in the same sample and by molecular dynamics simulation in a platinum nanowire^24^. These phenomenon was attributed to the discrete first order martensitic transformation which govern the elastic deformation behaivors of Nb nanowire. These discrete martensitic transformation leads to achievement of maximum elastic strain in a localized region in Nb nanowire adajacent to martensite plate, even though the external strain was only a fraction of the transformation strain and the a great fraction of NiTi remain to be austenite.

Figure 5 shows analysis of the strain distribution from the surface to the center of the nanowire. The lattice strains were measured from the green box, as shown in Fig. 5a, at an interval of ~1 nm along direction perpendicular to the surface. The lattice distance at each interval was calculated by averaging seven (110)Nb planes along the length direction. Figure 5b shows the strain distribution as a function of distance from the surface. The results revealed that the strain decreases gradually from the surface to the center of the nanowire. The distribution of strain inside the Nb nanowire as a function of distance from the tip of martensite is schematically illustrated in Fig. 5c. The strain measurement shown in Figs 4 and 5 indicate that the thicker the nanowire is, the smaller the strain that can be transferred from the NiTi matrix.

In this study, we directly observed the achievement of a maximum elastic strain of as high as 8%, close to the theoretical elastic strain limit of nanomaterials, in Nb nanowire embedded in NiTi matrix. The Nb nanowire exhibits localize and inhomogeneous elastic deformation, governed by the discrete martensitic transformation which produces lattice distortion strain fluctuation between the martensite and the austenite regions. Also because of the discrete martensitic transformation, the maximum elastic strain can be achieved at a small external strain, different from the monotonous increase of elastic strain with increasing external strain in bulk Nb/NiTi.

## Methods

A Ti_42_Ni_38_Nb_20_ (at.%) ingot was prepared by means of vacuum induction melting, followed by hot forging at 850 °C and hot drawing to a wire of 2 mm in diameter. The alloy wire contained Nb nanowires embedded in a NiTi(Nb) matrix. The matrix composition was determined by EDS analysis to be Ni:Ti:Nb = 50.6:46.1:3.3 in atomic ratio. The wire was then annealed at 550 °C for 30 min followed by air cooling. TEM specimens were first mechanically ground and then twin-jet electrochemically polished in an electrolytic solution of methanol - H_2_SO_4_ 14 vol.% at −20 °C. A small rectangular plate of about 1 × 0.2 mm^2^ was cut by means of focused ion beam (FIB) milling from a thin area near the electrochemically polished hole of the TEM sample. Careful procedure was taken to minimize the damage caused by ion milling to the sample. The FIB milling was first conducted under a bias voltage of 30 kV, followed by polishing (a very slow milling) under 2 kV to remove the damaged surface layers caused by the first fast milling. The thin NiTi plate was then transferred to a special TEM tensile apparatus designed for conducting *in situ* tensile deformation in TEM. The tensile deformation and TEM observation were conducted using a JEOL HRTEM with a field-emission gun (JEOL 2010 F) and a point resolution of 0.19 nm.

## Additional Information

**How to cite this article**: Zang, K. *et al.* Revealing ultralarge and localized elastic lattice strains in Nb nanowires embedded in NiTi matrix. *Sci. Rep.* **5**, 17530; doi: 10.1038/srep17530 (2015).

## Figures and Tables

**Figure 1:**
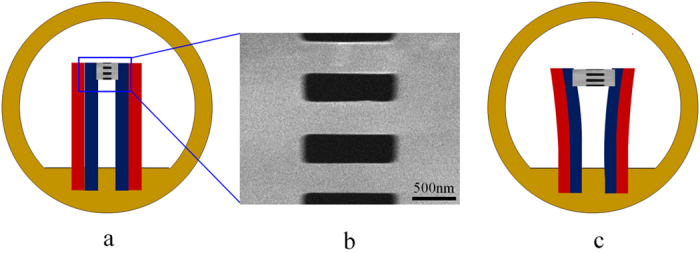
Schematic illustrations of the tension apparatus and sample design. (**a**) The tension apparatus composing two bimetallic arms fixed onto a *φ*=3 mm TEM copper grid (the dimensions are not in proportion). (**b**) The microbeam samples for *in situ* tensile testing cut from a microplate glued onto the tips of the bimetallic arms. (**c**) Schematic illustration of the tensile deformation induced by heating.

**Figure 2:**
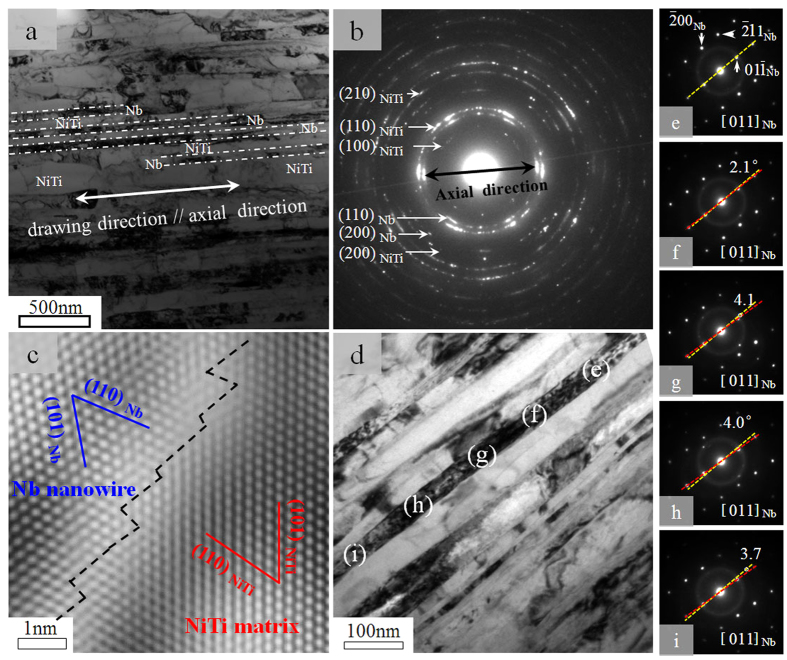
TEM study of the microstructure of the Nb/NiTi nanocomposite. (**a**) Bright field TEM image of a longitudinal section of the Nb/NiTi nanocomposite. (**b**) Selected area electron diffraction (SAED) pattern of the area shown in (**a**). (**c**) A high resolution TEM image of the Nb/NiTi interface. (**d**) Bright field TEM image of a region studying the orientation distribution along one individual Nb nanowire. (**e**–**i**) SEAD patterns of regions (**e**–**i**) identified in (**d**) along the nanowire.

**Figure 3:**
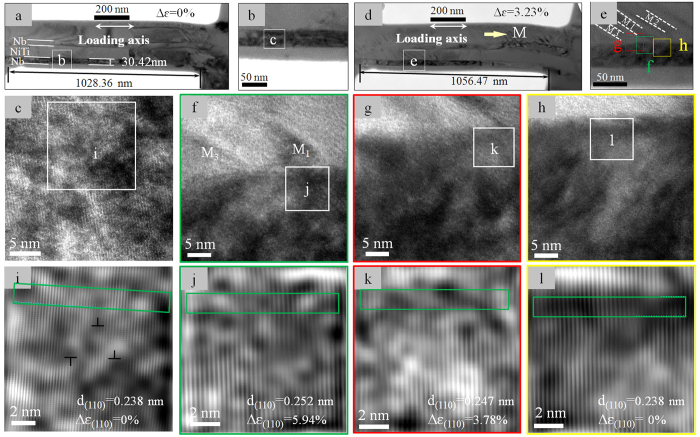
TEM study of lattice strain matching between Nb nanowires and the NiTi matrix. (**a**) Bright field image of a microbeam fabricated by means of focus ion beam milling. (**b**) Enlarged view of a Nb nanowire in the beam. (**c**) HRTEM image of the region marked as c in (**b**). (**d**) TEM image of the microbeam upon loading to ~3.23% strain. (**e**) Enlarged TEM image of the region marked as e in (**d**), showing the three martensite plates (marked as M_1_, M_2_ and M_3_) nucleated in the NiTi matrix. (**f**–**h**) HRTEM images of the regions marked as f, g and h in (**e**). (**i**–**l**) Filtered HRTEM images of the regions marked as i in (**c**) and as **j**-**l** in (**f**–**h**).

**Figure 4:**
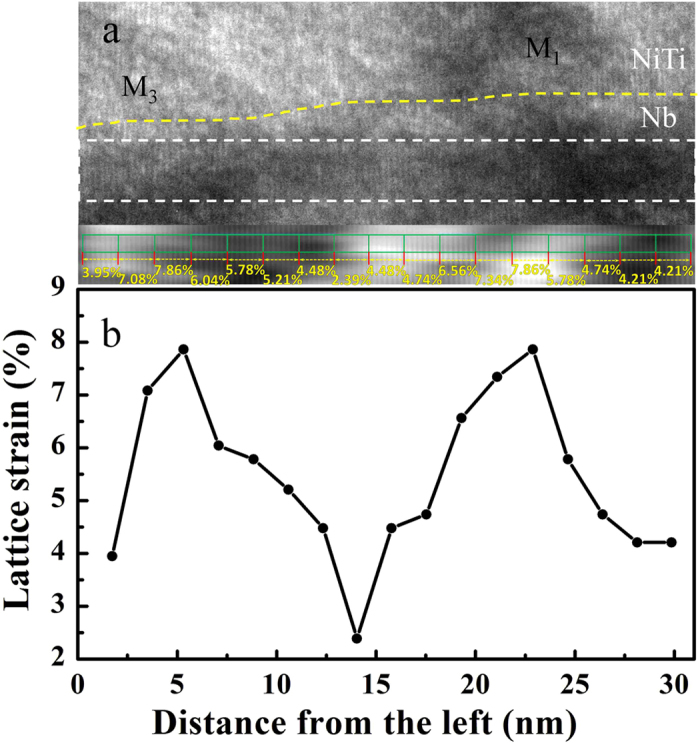
Measurement of the (110)_Nb_ lattice strain distribution along the Nb nanowire. (**a**) HRTEM image of the region covering martensite plates M1 and M3. The interplanar spacing variation was calculated at an interval of seven (110)_Nb_ planes. (**b**) Distribution of the lattice strain as a function of distance from the start plane.

**Figure 5:**
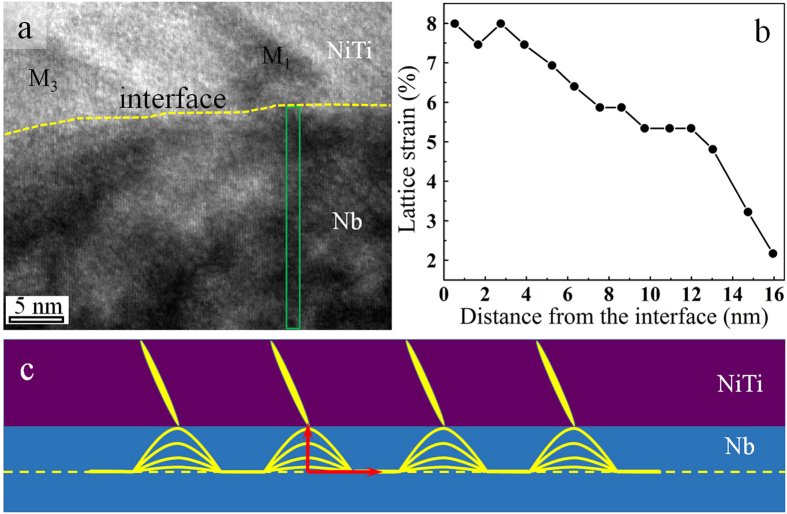
Measurement of the (110)Nb lattice strain distribution perpendicular to the Nb nanowire. (**a**) HRTEM image of the region covering martensite plates M1 and M3. The interplanar spacings were calculated at an interval of ~1 nm along the direction perpendicular to the interface. At each interval, the lattice distance was calculated by averaging seven (110)Nb planes along the length direction of nanowire. The measurement region is indicated using the green box. (**b**) Distribution of the lattice strain in the Nb nanowire length direction as a function of distance from the interface. (**c**) Schematic illustration of the lattice strain distribution inside the Nb nanowire. The horizontal coordinate represents the horizontal distance from the tip of martensitic plate, and the vertical coordinate represents the lattice strain in the nanowire length direction. Each arched curve represent a different vertical distance from the surface.
